# From genotype to phenotype: adaptations of *Pseudomonas aeruginosa* to the cystic fibrosis environment

**DOI:** 10.1099/mgen.0.000513

**Published:** 2021-02-02

**Authors:** Laura Camus, François Vandenesch, Karen Moreau

**Affiliations:** ^1^​ CIRI – Centre International de Recherche en Infectiologie, Université de Lyon/Inserm U1111/Université Claude Bernard Lyon 1/CNRS UMR5308/ENS de Lyon, Lyon, France; ^2^​ Centre National de Référence des Staphylocoques, Hospices Civils de Lyon, Lyon, France; ^3^​ Institut des Agents Infectieux, Hospices Civils de Lyon, Lyon, France

**Keywords:** adaptation, cystic fibrosis, genomic, phenotype, *Pseudomonas aeruginosa*

## Abstract

*
Pseudomonas aeruginosa* is one of the main microbial species colonizing the lungs of cystic fibrosis patients and is responsible for the decline in respiratory function. Despite the hostile pulmonary environment, *P. aeruginosa* is able to establish chronic infections thanks to its strong adaptive capacity. Various longitudinal studies have attempted to compare the strains of early infection with the adapted strains of chronic infection. Thanks to new ‘-omics’ techniques, convergent genetic mutations, as well as transcriptomic and proteomic dysregulations have been identified. As a consequence of this evolution, the adapted strains of *P. aeruginosa* have particular phenotypes that promote persistent infection.

## Data Summary

Supporting data are available in Table S1, available with the online version of this article.

**Impact Statement**

The chronic lung infections caused by *Pseudomonas aeruginosa* are associated with the deterioration of pulmonary functions and general health of cystic fibrosis (CF) patients. The difficulty of efficiently eradicating this pathogen comes from its ability to evolve towards high-persistence phenotypes through genetic adaptation. Understanding the basis and the determinants of this evolution is, thus, essential for the identification of new strategies to limit lung colonization by *P. aeruginosa *. The sequencing studies performed on CF isolates have highlighted numerous different evolutionary paths taken by the bacterium, leading to an intense intrapatient and interpatient diversification of *P. aeruginosa* populations. Fortunately, the identification of convergent patterns of adaptation is now possible thanks to the increasing number of research studies focused on CF isolates worldwide. Previous reviews on the topic often focused on particular aspects of *P. aeruginosa* adaptation, such as the genome dynamic, diversification processes or metabolism. In the present review, all the different aspects, as well as the latest publications on the topic, have been compiled to provide an updated and broader viewpoint of *P. aeruginosa* adaptation to the CF environment. This review also highlights convergent adaptation patterns involving intergenic regions, and transcriptomic and proteomic profiles of *P. aeruginosa *, not fully explored until now.

## Introduction

The ability of *Pseudomonas aeruginosa* to establish a chronic infection in cystic fibrosis (CF) lungs despite a wide range of stress sources highlights its high adaptability. In fact, the high plasticity of the *P. aeruginosa* core and accessory genome allows the bacterium to colonize a wide variety of environments, such as soils, water or abiotic surfaces [1–4]. However, *P. aeruginosa* adaptive processes have been especially described in the context of pulmonary infections. Indeed, the chronicity of *P. aeruginosa* CF lung infections and the difficulty in treating them make it essential to understand the mechanisms of the persistence. Moreover, this chronic infectious disease offers a rare opportunity to study long-term microbial evolution within a human host. The creation of CF centres has facilitated the conservation of the different micro-organisms isolated from CF patient sputa, allowing the constitution of longitudinal isolate banks from numerous subjects. This also contributed to the identification of highly transmissible *P. aeruginosa* strains such as the lineages DK2, AUST-02, LES (Liverpool epidemic strain) and C that are epidemic in Denmark, Australia and the UK, respectively [5]. Thanks to the development of next-generation sequencing methods, many studies have focused on longitudinal genetic adaptation of *P. aeruginosa* to the CF lung environment (Table 1). In 2006, Smith and colleagues were the first to describe a genetic evolution of a clonal lineage of *P. aeruginosa in vivo* by sequencing two *P. aeruginosa* strain isolates collected 7.5 years apart from the same patient [6]. Following studies were performed on a broader range of isolates from unique patients [7–12] or on transmissible lineages such as DK2 or AUST-02 [13–15]. Finally, Marvig *et al*. and Klockgether *et al*. combined both approaches to study the genomics of, respectively, 474 and 262 isolates from more than thirty patients [16–18].

**Table 1.** Genomic studies performed on longitudinal CF isolates of *P. aeruginosa
* The list of genes or intergenic regions identified in these studies was used to highlight the most mutated regions in Tables 2 and 3. The most representative genomic studies performed on *P. aeruginosa* CF sequential isolates whose isolations were spaced by at least 1 year were selected.

Sequencing type	No. of patients	No. of sequenced isolates	Time span of isolate evolution (years)	No. of studied lineages or clone types	Identification of positively selected genes	Reference
Whole-genome	1	2	7.5	1	No	[6]
Gene-targeted	29	58	5–20	nd	No	
Whole-genome	1	45	20	1 (PA14)	No	[7]
	1	63	23	1 (C)	No	
Whole-genome	6*	12*	35 max.*	1 (DK2)*	No	[13]*
Whole-genome	21*	55*	36*	1 (DK2)*	Yes	[14]*
Whole-genome	1	18	32	1 (DK1)	No	[9]
Whole-genome	1	13	6	1	Yes	[8]
	1	14	20	1		
Whole-genome	34	474	1–8	53 (36 for PE)	Yes	[16]
Whole-genome	4	26	17–19	6	Yes	[18]
Whole-genome	1	2	6.9	1 (OC4A)	No	[10]
Whole-genome	1	2	3	1	No	[12]
Whole-genome	32 (12 for PE)	262	<15–35	12	Yes	[17]
Whole-genome	13 (6 for PE)	63	3–4	1 (AUST-02)	Yes	[15]
Whole-genome	1	40	8	1	Yes	[11]
Reanalysis of whole- genome sequencing	68	534	nd	44	Yes	[81]

nd, Not determined in the study; PE, parallel evolution.

*Isolates sequenced by Yang *et al*. were also used in the study by Marvig *et al*., making the results of these two studies interconnected [13, 14].

**Table 2.** *
P. aeruginosa* genes identified as non-synonymously mutated in at least three independent longitudinal studies The characteristics of the 13 studies used for the intragenic regions are listed in Table 1.

				Longitudinal studies	
Gene name	PAO1 locus	Product	Positive selection	No.	Reference
*gyrB*	PA0004	DNA gyrase subunit B	Yes	8	[6, 7, 9, 11, 14–16, 18]
*pvdS*	PA2426	Sigma factor	No	8	[6, 7, 9, 10, 13, 16–18]
*mexA*	PA0425	RND multidrug efflux membrane fusion protein MexA precursor	No	6	[6, 7, 15–18]
*mexY*	PA2018	Multidrug efflux protein	No	6	[6–8, 10, 13, 14, 17]
*mexZ*	PA2020	Transcriptional regulator of multidrug efflux pump	Yes	6	[6, 9, 13, 16–18]
*gyrA*	PA3168	DNA gyrase subunit A	No	6	[9, 10, 13, 14, 16–18]
*ftsI*	PA4418	Penicillin-binding protein 3	No	6	[8–10, 13–15, 17]
*mexB*	PA0426	RND multidrug efflux transporter	No	6	[6, 7, 14, 16–18]
*oprD*	PA0958	Basic amino acid, basic peptide and imipenem outer-membrane porin	No	6	[9, 10, 14–17]
*migA*	PA0705	α-1,6-Rhamnosyltransferase	No	5	[7, 9, 12–15]
*algU*	PA0762	RNA polymerase sigma factor	Yes	5	[9, 13, 14, 16–18]
*lasR*	PA1430	Transcriptional regulator of QS	Yes	5	[6, 9, 16–18]
*pmrB*	PA4777	Two-component regulator system signal sensor kinase	No	5	[7, 10, 11, 13–15]
*mucA*	PA0763	Anti-sigma factor	Yes	5	[6, 13, 16–18]
*algG*	PA3545	Alginate-C5-mannuronan-epimerase	No	5	[7, 9, 12, 13, 17]
*mexS*	PA2491	Probable oxidoreductase	Yes	4	[6, 15–17]
*mexT*	PA2492	Transcriptional regulator of multidrug efflux pump	No	4	[6, 10, 12, 15]
*rpoB*	PA4270	DNA-directed RNA polymerase β chain	No	4	[6, 7, 13, 14, 17]
*chpA*	PA0413	Component of chemotactic signal transduction system	No	4	[7, 10, 11, 17]
*wbpM*	PA3141	Nucleotide sugar epimerase/dehydratase	No	4	[9, 10, 16, 17]
*fusA1*	PA4266	Elongation factor G	Yes	4	[8, 9, 12, 17]
*rpoN*	PA4462	RNA polymerase C-54 factor	Yes	4	[6, 13, 15, 18]
*pagL*	PA4661	Lipid A 3-*O*-deacylase	Yes	4	[9, 12, 13, 17]
*retS*	PA4856	Regulator of exopolysaccharide and type III secretion	No	4	[7, 10, 16, 18]
*rpoC*	PA4269	DNA-directed RNA polymerase subunit β	No	3	[7, 13, 14, 17]
*exsA*	PA1713	Transcriptional regulator of T3SS	No	3	[6, 7, 12]
*ampC*	PA4110	β-Lactamase/d-alanine carboxypeptidase	Yes	3	[8, 9, 13, 14]
*atsA*	PA0183	Arylsulfatase	No	3	[7, 10, 13]
*pilJ*	PA0411	Twitching motility protein	Yes	3	[9, 13, 17]
*xdhB*	PA1523	Xanthine dehydrogenase	No	3	[7, 8, 10]
*dnaX*	PA1532	DNA polymerase subunits γ and τ	No	3	[6, 10, 16]
*pcoA*	PA2065	Copper resistance protein A precursor	No	3	[7, 10, 16]
*pvdL*	PA2424	Non-ribosomal peptide synthase, pyoverdine biosynthesis	No	3	[9–11]
*clpA*	PA2620	ATP-binding protease component	Yes	3	[9, 13, 17]
*pelA*	PA3064	Glycohydrolase involved in Pel biosynthesis	No	3	[10, 14, 16]
*hasR*	PA3408	Haem uptake outer-membrane receptor precursor	No	3	[7, 10, 17]
*wspA*	PA3708	Chemotaxis transducer	No	3	[10, 16, 17]
*PA3728*	PA3728	ATPase	Yes	3	[8, 13, 17]
*purL*	PA3763	Phosphoribosylformylglycinamidine synthase	Yes	3	[8, 13, 17]
*bfmS*	PA4102	Histidine kinase sensor	No	3	[9, 12, 15]
*recC*	PA4285	Exodeoxyribonuclease V subunit γ	No	3	[7, 8, 10]
*ampD*	PA4522	*N*-Acetyl-anhydromuranmyl-l-alanine amidase	No	3	[6, 7, 17]
*nfxB*	PA4600	Transcriptional regulator	Yes	3	[16–18]
*phuR*	PA4710	Putative haem/haemoglobin uptake outer-membrane receptor	No	3	[7, 15, 17]
*cbrA*	PA4725	Two-component sensor CbrA	No	3	[10, 11, 17]
*cbrB*	PA4726	Two-component response regulator CbrB	No	3	[7, 9, 13]
*folP*	PA4750	Dihydropteroate synthase	No	3	[7, 10, 15]
*spoT*	PA5338	Guanosine-3',5'-bis(diphosphate) 3'-pyrophosphohydrolase	Yes	3	[9, 13, 17]

**Table 3.** Selection of *P. aeruginosa* intergenic regions under positive selection Mutations in intergenic regions were identified as positively selected by Khademi *et al*. [81] and selected for this table according to their number, the number of affected lineages and the number of longitudinal studies highlighting mutations in the same intergenic region. The complete list is shown in Table S1.

Upstream/downstream genes		Upstream/ downstream PAO1 locus		Upstream/downstream products	No. of intergenic mutations	No. of lineages	Reference
phuS // phuR		PA4709 // PA4710		PhuS/haem/haemoglobin uptake outer-membrane receptor	40	4	[7, 9, 18, 81]
PA0428 // ***PA0429***		PA0428 // PA0429		Probable ATP-dependent RNA helicase/hypothetical protein	34	10	[81]
***PA4786 // PA4787***		PA4786 // PA4787		Probable short-chain dehydrogenase/probable transcriptional regulator	28	12	[81]
PA4690.5 ***// PA4691***		PA4690.5 // PA4691		16S ribosomal RNA/hypothetical protein	54	6	[81]
***PA2535 // PA2536***		PA2535 // PA2536		Probable oxidoreductase/probable phosphatidate cytidylyltransferase	18	6	[7, 81]
motY ***//*** pyrC		PA3526 // PA3527		Probable outer-membrane protein precursor/dihydroorotase	32	6	[81]
PA3230 ***// PA3231***		PA3230 // PA3231		Conserved hypothetical protein/conserved hypothetical protein	24	7	[81]
***algL** //* algl		PA3547 // PA3548		Poly(β-d-mannuronate) lyase precursor/alginate *O*-acetyltransferase	14	6	[7, 81]
***PA0976.1 // PA0977***		PA0976.1 // PA0977		tRNA-Lys/hypothetical protein	26	6	[81]
rplU *//* ispB		PA4568 // PA4569		50S ribosomal protein L21/octaprenyldiphosphate synthase	22	7	[81]
phzM *//* phzA1		PA4209 // PA4210		Probable phenazine-specific methyltransferase	12	6	[7, 81]
oprO *// **PA3281***		PA3280 // PA3281		Pyrophosphate-specific outer-membrane porin precursor/hypothetical protein	10	5	[7, 81]
ldh *//* PA3419		PA3418 // PA3419		Leucine dehydrogenase	10	5	[7, 81]
ampR *//* ampC		PA4109 // PA4110		Transcriptional regulator/β-lactamase precursor	12	4	[9, 81]
PA5160.1 ***// rmlB***		PA5160.1 // PA5161		tRNA-Thr/dTDP-d-glucose 4,6- dehydratase	16	6	[81]

Genes of which the promoter is located in the impacted intergenic region are underlined.

By gathering the results of these different longitudinal studies, we aim to provide an updated description of the main genetic adaptations of *P. aeruginosa* to the CF lung environment. In this review, we will also discuss how these alterations affect transcriptomic and proteomic profiles of *P. aeruginosa* thanks to the latest studies performed on clinical CF isolates. Finally, common phenotypes of CF-adapted *P. aeruginosa* will be described.

## Genomic adaptation of *P. aeruginosa*

### *P. aeruginosa* genome accumulates mutations during establishment of chronic colonization

#### Types and frequency of mutational events

Longitudinal genomic studies highlighted that late isolates of *P. aeruginosa* present numerous genetic modifications in comparison to early isolates. Small mutational events such as SNPs or short insertions and deletions (indels) have been described as the major driver of these modifications. Indeed, the *P. aeruginosa* genome was shown to accumulate a median of 3 SNPs per year, varying between 0.5 and 14 SNPs per year [6–9, 12, 14–18]. Small indels have also been reported at rates ranging from 0.4 to 2.7 indels per year (0.1 to 0.28 indels per SNP) [12, 14, 17]. These modifications could be observed on both core and accessory genomes of clinical isolates, depending on the use of a reference strain for gene annotation. Indeed, while several studies focused on the annotated genes in PAO1 or PA14 [6, 7, 13, 16, 17], others were able to identify SNPs in clone-specific genes using a related ancestral isolate as a reference [8, 9, 12, 14, 17]. The presence of accessory elements such as genomic islands and prophages could also be predicted *in silico* [10, 11].

The role of the accessory genome is in fact increasingly considered for understanding *P. aeruginosa* adaptive processes, due to its plasticity and the richness of its encoded functions [4, 19]. Indeed, the *P. aeruginosa* pathogenic islands (PAPIs) and several LES prophages were shown to affect diversification processes and important pathoadaptive phenotypes of *P. aeruginosa *, including its ability to establish *in vivo* and its antibiotic resistance [20–25]. Such elements can be horizontally transferred between *P. aeruginosa* or even between different microbial species through mechanisms of phage infection or pilus-mediated conjugation of excised and circularized genomic islands [4, 26–30]. However, acquisition of novel DNA through horizontal gene transfer remains rare [31, 32] and the genome of *P. aeruginosa* rather tends to shrink during its adaptation in CF lungs. Rau *et al*. described that the *P. aeruginosa* DK2 lineage underwent a loss of a mean of 4.2 kbp per year [31]. Deletions of more than 1000 bp have been observed in other lineages (in 10 out of 12 lineages in the study of Klockgether and colleagues), with the size of deleted regions reaching 188 kb [6, 7, 11, 17]. Here again, these deletions were shown to affect both core and accessory genomes, as prophages and genomic islands were shown to be partially or totally lost during *P. aeruginosa* adaptation to the CF environment [11, 27, 28, 31, 33–35]. Notably, the genomic islands PAPI-1 and the *P. aeruginosa* genomic island-2 (PAGI-2) were found either excised or impacted by deletions in CF isolates [27, 28]. In contrast, other elements of the accessory genome seem less prone to deletions, as the toxin–antitoxin systems, the clustered regularly interspaced short palindromic repeats (CRISPR) spacers and the genomic island PAGI-1 are well conserved in CF isolates [36–38].

In addition to deletions, the *P. aeruginosa* genome can undergo important chromosomal rearrangements that often involve accessory mobile elements, such as transposons and integrons [4, 19]. The insertion sequence IS*6100* was identified as the main perpetrator of the frequent chromosomal inversions observed in the CF strains from clone C [35, 39]. Besides disrupting the reading frame of neighbouring genes [39], such chromosomal rearrangements can have pleiotropic consequences through modifications of regulatory regions or DNA topology [40]. By assessing the phenotype–genotype relationship of 44 isolates from a single patient, Darch *et al*. highlighted that the phenotypic diversity observed between CF isolates was mainly due to homologous recombination mechanisms [41]. However, this result and the high recombination rate obtained were then shown to mainly arise from false-positive events. New bio-informatics analyses of the same sequencing data with correcting filters indeed indicated lower recombination rates [42, 43]. These discrepancies emphasize the importance of bio-informatics tools and settings for the identification of recombination events, and more broadly for all genomic comparisons. In that respect, the detection of genetic alterations can be improved by combining second- and third-generation sequencing methods: while second-generation sequencing such as Illumina provides short reads with low error rates, the longer reads generated by third-generation sequencing allow a better detection of recombination events and large chromosomal rearrangements.

#### Hypermutability

The rate of spontaneous mutations can be affected by the genetic background of the strain, and even enhanced by previous mutational events. For instance, the high rates of deletion observed by Rau *et al*. can be attributed to stochasticity or to the presence of missense mutations in the coding sequences of the exonucleases *sbcB* and *sbcC* implicated in recombination [31]. In the same way, the well-known hypermutable phenotype of *P. aeruginosa* arises from genetic alterations of DNA repair systems. Indeed, mutations in *mutS/mutL* and *uvrD* genes are commonly observed in CF isolates and induce a significant increase of the mutation rate [44]. Chromosomal inversions were also shown to disrupt the reading frame of *mutS* and induce hypermutability in clinical strains from the C lineage [39]. Hypermutable isolates, thus, accumulate a mean of 16-fold more mutations, with a median of 48 SNPs per year (range of 2 to more than 350 SNPs per year) [7, 8, 15, 17, 45].

Hypermutability increases the genetic diversity of the *P. aeruginosa* population in CF lungs, an advantageous feature for adaptability to stressful conditions [8, 14, 16, 17, 46, 47]. Indeed, it has been shown that antibiotic exposure promotes the emergence of hypermutability in *P. aeruginosa *, then favouring acquisition of antibiotic resistance [45, 48–51]. However, Mehta and colleagues also observed that some hypermutable lineages would spontaneously decline and disappear from the evolving population [49]. This phenomenon could be explained by an accumulation of neutral and/or slightly deleterious mutations whose probability is also increased by hypermutability. Moreover, the fitness benefit of hypermutators seems to be restricted to the conditions in which they evolved, as the accumulated hitchhiking mutations can constitute a burden in non-selective conditions [49, 50]. Hypermutability is, thus, a double-edged sword that does not ensure the success of *P. aeruginosa* adaptation. Indeed, hypermutators rarely dominate the colonizing population and coexist with normo-mutable isolates in CF lungs, potentially through colonization of specific niches [8, 9, 14]. Compensation of the hypermutator phenotype through secondary mutations has also been reported during adaptation to CF environment [8], suggesting an importance of the phenotype at certain stages of evolution. This hypothesis is supported by the high prevalence of hypermutators in CF cohorts. Since the first estimations by Oliver [44], several studies in European and American cohorts confirmed that a mean of 28 % of CF patients were infected by at least one hypermutable isolate of *P. aeruginosa* [44, 52–55]. Finally, despite a high prevalence and an increased ability to develop antibiotic resistance, the impacts of infection by hypermutable *P. aeruginosa* on clinical outcome are unclear. While an association between the presence of hypermutators and the deterioration of lung function was described in English and French cohorts [56, 57], such a result was not confirmed in an Israeli cohort [55]. Moreover, Klockgether and colleagues did not highlight a correlation between annual rate of sequence variation and the severity of the clinical course of German CF patients [17].

### Accumulation of mutations relies on selection mechanisms

The accumulation of mutations in the *P. aeruginosa* genome could be the result of genetic drift or neutral selection, during which mutations are stochastically fixed regardless of their impact. However, due to the stressful conditions inherent to the CF lung environment, mutations are actually selected because of their beneficial effect on bacterial fitness. As non-synonymous mutations are more likely to affect protein function and eventually fitness, selective mechanisms can be quantified by the non-synonymous to synonymous mutations ratio (*d*_N_/*d*_S_). This ratio can be calculated over different scales – from all coding regions of the pangenome to specific coding regions. Three type of selective mechanisms, thus, can be observed: (i) a *d*_N_/*d*_S_ value over one testifies to positive selection, (ii) a value under one indicates purifying or negative selection, and (iii) a close to one ratio depicts typical genetic drift.

These three selective mechanisms have been observed for the *P. aeruginosa* genome during adaptation to the CF environment. Several studies have highlighted positive selection mechanisms at the genome scale (*d*_N_/*d*_S_ of 1.4 and 2) [6, 12], whereas negative selection was observed in others (*d*_N_/*d*_S_ between 0.33 and 0.79) [7–9, 14]. In fact, selective mechanisms appear to vary according to the colonization time and clinical status of patients, affecting the accumulation of mutations and the composition of the accessory genome. Klockgether and colleagues observed that the *P. aeruginosa* genome presented *d*_N_/*d*_S_ ratios ranging from 0.39 to 1.66 according to the colonization time, mutability of isolates and the severity of infection [17]. A fluctuation of positive, neutral and negative selections with time was depicted for hypermutable strains causing severe and mild infections, and for normo-mutable isolates from mildly affected patients. Interestingly, only genomes of normo-mutable isolates from patients with severe infection presented a signature of positive selection during almost all the course [17]. A relationship between the severity and the accessory genome was also observed as isolates causing severe and mild infections presented divergent repertories of accessory genes. Similar observations were previously made for persistent and eradicated CF isolates [17, 32]. In addition, Cramer *et al*. and Markussen *et al*. observed a rapid genetic diversification during the first clades followed by coexistence of more stable sublineages of PA14 and DK1, respectively [7, 9]. Similarly, the DK2 lineage was shown to have accumulated most mutations before 1979 in order to ensure its success in several hosts, after which negative selection was observed [13]. In both studies, late *P. aeruginosa* isolates tended to accumulate fewer mutations than early ones, suggesting modifications of selection mechanisms over the time [7, 9, 11, 13]. Mutations are indeed less likely to improve fitness and, thus, to be fixed once *P. aeruginosa* is adapted to the CF environment. Compensation of the hypermutable phenotype by secondary mutations observed by Feliziani and colleagues [8] supports this notion, as it can rebalance the mutation rate to a regular level after a stage of rapid diversification and adaptation. Finally, several recent research studies on non-CF infections reported that *P. aeruginosa* adaptive mechanisms occur at the very beginning of the colonization, emphasizing the underappreciated role of genetic adaptation in acute infections [51, 58, 59]. Altogether, these results indicate that different modes of selection arise with time, according to infection stage and severity. Thus, we suggest that positive selection first occurs during acute infections, which often severely affect patient clinical status. Thereafter, neutral or negative selection is promoted as *P. aeruginosa* adapts and the infection becomes chronic.

Although general trends of positive or negative selection can be observed for the global genome, it is important to note that selection can vary considerably according to the DNA segment. Thus, genes from the antibiotic resistome can appear positively selected despite negative selection at the genome scale [8, 45]. In contrast, negative selection is particularly depicted in the accessory genome of *P. aeruginosa *, where loss of DNA and accumulation of synonymous SNPs are promoted by mutational hotspots and genomic instability [6, 10, 31, 32, 60, 61]. However, the negative selection in accessory segments compared to the core genome can sometimes be offset by DNA acquisition through horizontal gene transfer, as described in the clones C and PA14 [10, 60]. Finally, the genetic background of *P. aeruginosa* can also influence selection and fixation of mutations in particular genes through epistatic mechanisms. Certain genetic alterations, thus, may be positively selected due to their compensatory effect on former polymorphisms or in a given genetic background, as depicted in several cases. Damkiaer and colleagues observed that a single *rpoD* mutation induced alginate overproduction only in a particular genetic background of the DK2 lineage and, thus, was positively selected [62]. Genic alterations of *mexT* were shown to compensate the effects of *lasR* inactivation, suggesting that positive selection of this mutation may be promoted in *lasR-*negative isolates [63–66]. In the same way, mutations reverting the mutator phenotype might be positively selected only after alteration of the genes from DNA repair systems [8].

Besides colonization time, infection severity and the genetic background of isolates, spatial isolation can affect the dynamics of selection mechanisms. Indeed, it is now well understood that micro-organisms can be subject to highly different selective pressures according to the environment. The heterogeneity of the CF lung ecosystem generates ecological microniches with variable physicochemical and biotic characteristics and, thus, variable selective forces. As a result, a phenomenon of adaptive radiation can be observed during *P. aeruginosa* adaptation to the CF environment. Divergent evolutionary patterns have indeed been depicted between clonally related isolates that have evolved in sinuses or in lungs [9], and even between clones isolated from different lung regions [67]. In both studies, isolates evolved independently within the different regions, as no phenomenon of convergent evolution could be observed. Instead, genotypic and phenotypic diversification was shown to be driven by the spatial isolation of strains [9, 67]. This diversification leads to the coexistence of numerous clonal lineages in the CF airways, as excellently reviewed by Winstanley and colleagues [46].

In addition to this intra-clonal diversification, the heterogeneity of *P. aeruginosa* populations is promoted by the coexistence of several lineages within the lungs of CF patients. Thus, from a single sputum sample, different *P. aeruginosa* lineages are frequently isolated that were independently acquired from the environment or from other CF patients, especially for LES-derived lineages [46, 68, 69]. Williams and colleagues observed that the prevalence of each lineage within a patient was highly dynamic during the course of infection, affecting considerably the diversification processes of *P. aeruginosa* [69]. On the one hand, the lung colonization by divergent lineages was shown to bring more genetic diversity than the *in situ* evolution of *P. aeruginosa *. On the other hand, competition between lineages appeared to select for particular genotypes and, thus, influence the diversification processes of *P. aeruginosa *. In a CF patient, the replacement of a LES lineage by another, thus, could be associated with an increased frequency of pathoadaptive mutations in the *lasR* gene [69]. The other way round, one would also expect that the presence of certain genotypes within lungs can either promote or limit superinfection by other *P. aeruginosa* lineages and, thus, interclonal diversification. This phenomenon can be extended to the colonization by other microbial species, as they have to cope with heterogeneous, adapted and niche-specialized populations of *P. aeruginosa *.

This genetic and phenotypic diversification of *P. aeruginosa* raises important issues concerning the sampling and the study of bacterial colonies from CF expectorations: a single colony is not representative of the infecting *P. aeruginosa* metapopulation [46]. In the case of longitudinal genomic studies, the sequencing of a single strain per time point is an important limitation and provides only a restricted fraction of the different evolutionary paths that the bacterium has taken. This issue obviously feeds through to all genotypic and phenotypic characterizations of CF *P. aeruginosa* strains, but is increasingly taken into account for sequencing studies and the determination of antibiotic-resistance profiles [34, 45, 68, 69].

### CF-adapted *P. aeruginosa* present pathoadaptive mutations

#### Coding regions

Despite the diversification processes of *P. aeruginosa *, the high number of genomic studies (Table 1) performed on sequential isolates allowed the identification of convergent patterns of adaptation. In addition to the *d*_N_/*d*_S_ calculation, genes under positive selection were brought out through different approaches: Marvig and colleagues determined genes that accumulated more mutations than what would be predicted if mutations were randomly distributed across the genome [14, 16, 18]. In other studies, thresholds were set to establish lists of genes that were hit by a minimum quantity of independent mutations and/or in a minimum number of lineages [8, 11, 17].

**Fig. 1.**
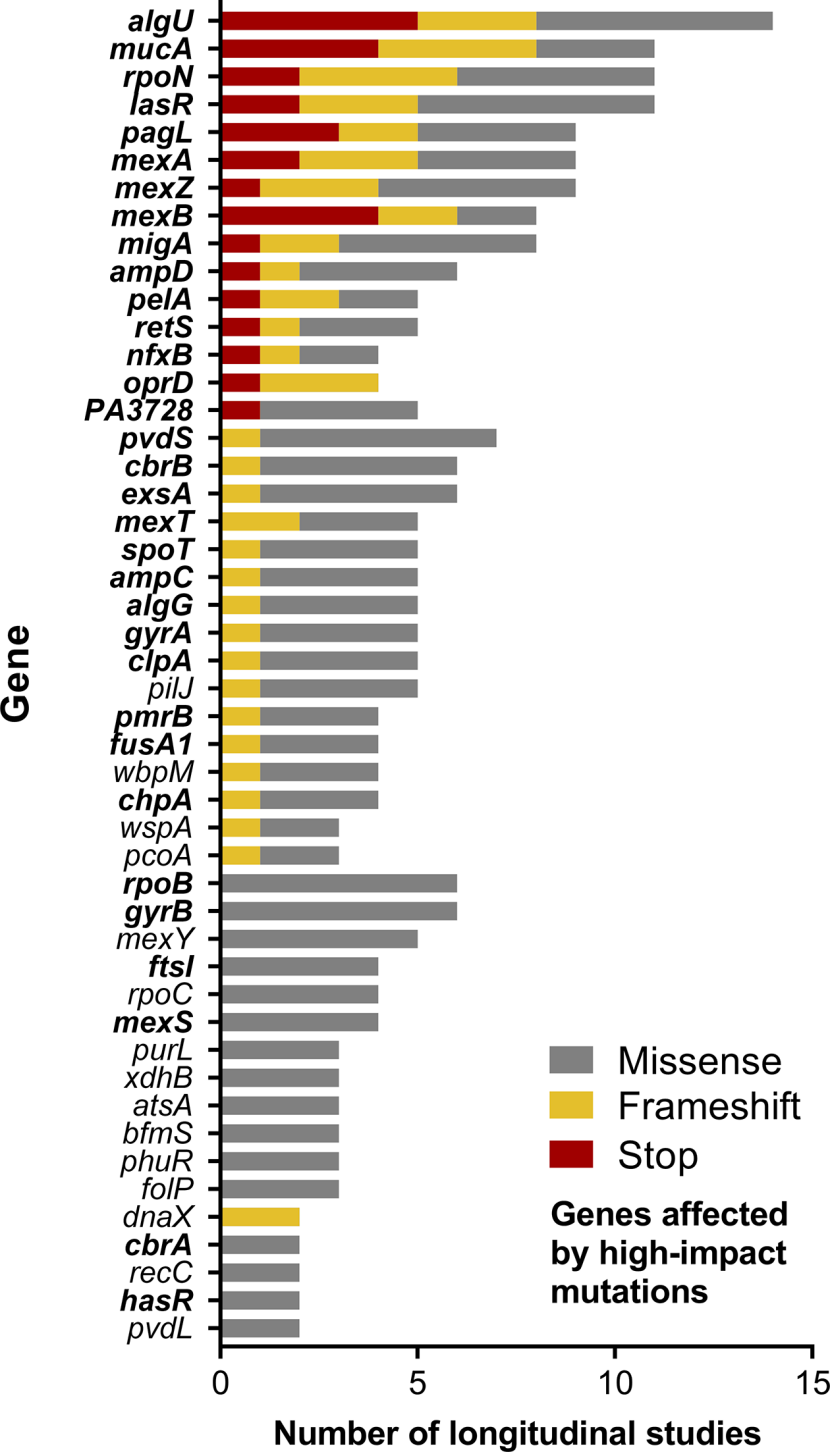
Number of longitudinal studies identifying stop (red), frameshifts (yellow) or missense (grey) mutations in 48 genes. Non-synonymously mutated genes and corresponding types of mutations were recovered from the longitudinal studies listed in Table 1. Genes in bold were affected by mutations predicted to have a drastic impact on protein function [17] or induce a partial or total loss-of-function [6].

In order to have a global overview of the mutated genes during *P. aeruginosa* adaptation, the results of 13 longitudinal studies were examined (Table 1). Table 2 provides a list of 48 *P *. *aeruginosa* coding regions that have been identified as non-synonymously mutated in at least three of these studies. Different types of mutations, thus, were highlighted (missense, frameshift and stop), but their impacts also rely on their position in the gene. Despite the change of a single amino acid, missense mutations can indeed have drastic consequences on translation efficiency or protein function, especially when they affect important functional domains [6, 17, 63]. Missense mutations were notably predicted to drastically affect the protein function of RpoB and GyrB [17], or even induce total loss-of-function of MexS [6] (Fig. 1).

Nonsense mutations and frameshifts induced by insertions and deletions are predicted as high-impact mutations as they induce a disruption and/or an interruption of translation. Most of the genes described in Table 2 have been shown to accumulate high-impact mutations during *P. aeruginosa* adaptation during longitudinal studies (Fig. 1). It is especially the case for numerous global regulators, such as *mucA*, *algU*, *rpoN* and *lasR*, but also regulators related to antibiotic resistance (*nfxB*, *mexZ*) or type III secretion (*retS*, *exsA*).

The role of these genes in *P. aeruginosa* adaptation to the CF environment was confirmed in larger cohorts of clinical isolates, but through a wide variety of mutations. In that respect, 173 unique *lasR* variants have been detected by gene-targeted sequencing of 2583 CF isolates, with most of them inducing a loss of function [63]. Mutations in mucoidy related genes have also been researched in *P. aeruginosa* isolates from CF patients [70–73]. A recent study in a Brazilian cohort identified 30 new mutations in the *algUmucABD* operon and confirmed the high frequency of the *mucA22* mutation, inducing a premature stop codon in the *mucA* gene [74]. However, it is noteworthy that high-impact mutations do not inevitably induce a complete loss of function. Feltner and colleagues indeed observed a retained LasR activity in 25 % of cases despite missense or even nonsense mutations in the *lasR* sequence [63]. Similarly, *P. aeruginosa* strains carrying the nonsense *mucA22* mutation were recently shown to respond highly differently than Δ*mucA* mutants to acidified nitrite conditions [75]. These results highlight the complexity of fully evaluating the consequences of mutations on protein features, even for ones predicted to induce a drastic impact or a loss of protein function. It is particularly the case for global transcriptomic regulators as their alteration, however small, can affect the expression and function of numerous other genes.

Synonymous mutations can also have beneficial or detrimental impacts on fitness through alteration of protein folding, translation efficiency and rate [76, 77]. Adaptive synonymous mutations with an associated gain of fitness have been highlighted during experimental evolution of *Pseudomonas fluorescens* [78, 79]. Thus, it would not be surprising that synonymous mutations also contribute to *P. aeruginosa* adaptation in CF lungs, although their impact is still rarely considered.

#### Intergenic regions

None of the previous studies assessed whether positive selection also occurred in non-coding regions, although intergenic mutations were identified. Recently, an analogous ratio to *d*_N_/*d*_S_ was described to assess selective mechanisms occurring in non-coding regions, where *d*_N_ is replaced by the number of intergenic SNPs per intergenic site (*d*_I_) [80]. Even though this method has not been used on a *P. aeruginosa* genome yet, the signature of purifying selection was observed for intergenic sites of other species such as *Escherichia coli* or *Staphylococcus aureus* [80, 81]. However, Khademi and colleagues reanalysed the sequencing data of intergenic regions from several longitudinal *P. aeruginosa* genomic studies [6, 14, 16] and were able to establish a list of adaptive non-coding regions mutated in at least 3 of the 44 studied lineages [81] (Table 3).

Interestingly, some of the adaptive intergenic regions identified have been found to be mutated in other longitudinal studies of which the sequencing data were not used in the analysis by Khademi *et al*. [7, 9, 18], supporting their role in *P. aeruginosa* adaptation. Table 3 presents the 15 adaptive intergenic regions most frequently mutated, i.e. regions that accumulated the highest number of mutations, in the most elevated number of lineages and longitudinal studies. The complete table is shown in Table S1. Mutations in the *phuS*/*phuR* intergenic region were identified in the largest number of studies and at significant rates. Finally, we notice that mutations occurred in the intergenic region between *ampR* and *ampC*, a gene that was also identified as pathoadaptive (Table 2). Genetic modifications of intergenic regions, thus, appear to also play a role in *P. aeruginosa* adaptation to the CF environment, potentially through the transcriptomic dysregulation of surrounding genes [81].

It is noteworthy that the sequencing results of the 13 longitudinal studies analysed in this review could be connected thanks to the genomic annotations from the reference strains PAO1 or PA14. Thus, Tables 2 and 3 are not representative of the numerous mutations occurring within genes or intergenic regions specific to clinical isolates. Moreover, the accessory genome of *P. aeruginosa* presents very divergent profiles according to the isolates, with great variations in its composition and its organization [4, 19]. As a result, accessory elements present a higher sequence diversity [27, 32, 60, 82], limiting the establishment of convergent evolutionary patterns within the accessory genome. Nonetheless, it needs to be kept in mind that some accessory genes can have homologous functions other than those present in the core genome [4, 19] and, thus, sometimes compensate a mutation in a conserved gene.

## Phenotypical signatures of CF-adapted *P. aeruginosa*

### *P. aeruginosa* adapts its expression profiles to the CF environment

#### Gene expression

The comparison of transcriptomes or proteomes of sequential clinical isolates seems to be the most suitable for assessing impacts of *P. aeruginosa* adaptation on global expression profiles. Several longitudinal studies indeed performed transcriptional profiling and observed differences of global transcript abundance between early and late isolates [9, 13], but also on specific expressed genes [12, 13, 83–85]. Table 4(a) lists 41 *P *. *aeruginosa* genes differentially expressed between early and late CF isolates. Convergent patterns of expression could be identified *in vitro* in late isolates in comparison to related early isolates, for instance, a down-regulation of genes involved in secretion (Hcp secretion island I), the pseudomonas quinolone signal (PQS) and phenazine biosynthesis. Interestingly, more than a half of dysregulated genes presented in Table 4 have been shown to be part of RpoN, AlgU or LasR regulons, underscoring their significance in *P. aeruginosa* adaptive mechanisms [86–89].

**Table 4.** *
P. aeruginosa* transcriptomic alterations during adaptation to the CF lung environment Square colour indicates gene expression: up-regulation (red), down-regulation (green), undetermined (light grey), divergent according to studies (dark grey). (a) Gene expression in late isolates in comparison to related early isolates of *P. aeruginosa *. The 41 genes with a convergent pattern identified in at least four isolates were selected [12, 13, 83–85]. (b) Gene expression in clinical CF isolates *in vivo* (CF sputum, explanted lungs or zebra fish infection) in comparison to growth *in vitro* [91–94]. (c) Gene expression in PAO1 *in vivo* (murine infection model of acute pneumonia) in comparison to growth *in vitro* [95].

Gene name	PAO1 locus	Product	(a) Expression in late isolates	(b) Expression in CF isolates *in vivo*	(c) Expression in PAO1 *in vivo*			
***PA1323***	**PA1323^f^**	**Hypothetical protein**						
***PA1324***	**PA1324^f^**	**Hypothetical protein**						
***PA1471***	**PA1471**	**Hypothetical protein**						
*PA1559*	PA1559	Hypothetical protein						
***PA1592***	**PA1592**	**Hypothetical protein**						
*mexX*	PA2019	RND multidrug efflux membrane fusion protein						Key
***PA2485***	**PA2485**	**Hypothetical protein**						Up-regulation
*PA3691*	PA3691^g^	Hypothetical protein						Down-regulation
***lptF***	**PA3692^g^**	**Lipotoxon F**						Undetermined
***PA3819***	**PA3819**	**Conserved hypothetical protein**						Divergent
***osmE***	**PA4876**	**Osmotically inducible lipoprotein**						
***PA4880***	**PA4880**	**Probable bacterioferritin**						
***PA5212***	**PA5212**	**Hypothetical protein**						
***PA0045***	**PA0045**	**Hypothetical protein**						
*PA0046*	PA0046	Hypothetical protein						
*PA0047*	PA0047	Hypothetical protein						
***tagQ1***	**PA0070^a^**	**TagQ1**						
*pppA*	PA0075^a^	PppA						
*tagF1*	PA0076^a^	Hcp secretion island I (HSI-I) T6SS						
***icmF1***	**PA0077^a^**	**Hcp secretion island I (HSI-I) T6SS**						
*tssL1*	PA0078^b^	Hcp secretion island I (HSI-I) T6SS						
*tssK1*	PA0079^b^	Hcp secretion island I (HSI-I) T6SS						
*tssJ1*	PA0080^b^	Hcp secretion island I (HSI-I) T6SS						
*ttsA1*	PA0082^c^	Hcp secretion island I (HSI-I) T6SS						
*ttsB1*	PA0083^c^	Hcp secretion island I (HSI-I) T6SS						
*ttsC1*	PA0084^c^	Hcp secretion island I (HSI-I) T6SS						
***hcp1***	**PA0085**	**Hcp secretion island I (HSI-I) T6SS**						
*tagJ1*	PA0086^d^	Hcp secretion island I (HSI-I) T6SS						
*tssE1*	PA0087^d^	Hcp secretion island I (HSI-I) T6SS						
*tssG1*	PA0089^d^	Hcp secretion island I (HSI-I) T6SS						
***clpV1***	**PA0090^d^**	**ClpV1**						
*pqsC*	PA0998^e^	β-Keto-acyl-acyl-carrier protein synthase						
*pqsD*	PA0999^e^	Acetyl CoA ACP transacetylase						
*phnA*	PA1001	Phenazine biosynthesis protein						
*HsiB2*	PA1657	Conserved hypothetical protein						
*hcnA*	PA2193	Hydrogen cyanide synthase						
*tse5*	PA2684	Cell wall/membrane/envelope biogenesis						
*PA3021*	PA3021	Hypothetical protein						
***PA3729***	**PA3729**	**Conserved hypothetical protein**						
*cytN*	PA4133	Cytochrome *c* oxidase subunit						
***PA4317***	**PA4317**	**Hypothetical protein**						

Genes annotated with an identical letter belong to the same operon.

Genes in bold respond to the following criteria: (i) convergent expression in CF late isolates in comparison to early ones, (ii) convergent expression *in vivo* in comparison to *in vitro* growth,and (iii) specific dysregulations *in vivo* in comparison to PAO1.

It is important to remember that gene expression relies highly on growth conditions and that *in vitro* patterns are not necessarily representative of what happens *in vivo*. Thanks to the advance of transcriptomic methods, recent studies evaluated *P. aeruginosa* global gene expression ‘*in vivo*’, i.e. directly on clinical populations within sputum [90–92], ex-planted lungs from CF patients [93], or during non-human infection models [94]. Transcriptomic patterns induced by *in vivo* conditions are presented in Table 4(b). Comparable transcriptomic dysregulations to those observed for *in vitro* transcriptomic analyses were depicted for more than a half of the genes listed in Table 4(a, b), including the down-regulation of genes from the Hcp secretion island. Interestingly, these dysregulations seem to be specific to CF clinical isolates. The PAO1 reference strain, not adapted to the CF-environment, was shown to present a very divergent, if not opposite, transcriptomic pattern during *in vivo* infection. These results were nonetheless obtained using a murine model of acute pneumonia and should be confirmed in a chronic infection context [95] (Table 4c). Altogether, these transcriptomic studies underscored the role of several genes in *P. aeruginosa* adaptation to the CF environment due to: (i) convergent expression in CF-adapted isolates in comparison to non-adapted ones, (ii) convergent expression *in vivo* in comparison to *in vitro* growth, and (iii) specific dysregulations *in vivo* in comparison to PAO1. Genes meeting these three criteria are highlighted in Table 4.

#### Protein expression

*P. aeruginosa* protein expression during CF infections was mainly assessed by evaluating proteomic changes between clinical and reference strains or under certain conditions, as reviewed by Hare and Cordwell and by Kamath *et al*. [96, 97]. More recently, this approach was used to evaluate proteome responses of a set of clinical isolates cultivated under different conditions of nutrient and oxygen availability [98–100]. Clinical *P. aeruginosa* isolates presented a distinct proteome profile from PAO1, with convergent expression of many proteins despite a high genomic and phenotypic diversity between isolates. An over-expression of proteins involved in amino acid biosynthesis or drug resistance, with the example of MexY was specifically noted for clinical isolates [98, 99]. Several proteins involved in motility, chemotaxis and adhesion features were also down-regulated, including proteins from the Fli and Pil systems, confirming previous observations [96, 97].

To our knowledge, differences of the global proteome between early and CF-adapted clonal isolates of *P. aeruginosa *, however, have not been assessed yet, limiting the establishment of direct relationships between genetic adaptation to the CF environment and protein expression. Nonetheless, a recent study described the *P. aeruginosa* proteome directly from CF sputum. By comparing protein expression in the *P. aeruginosa* population from 35 samples, Wu and colleagues, thus, were able to identify a convergent pattern of protein expression *in vivo* [101] (Table 5a). Some of the proteins identified as more abundantly produced by clinical isolates than by PAO1 were found also to be highly produced *in vitro*, with the example of the chaperone Hfq and the phosphate transporter PtsS (Table 5b) [98, 99, 101–103]. Here again, protein expression pattern appears to largely rely on growth conditions (Table 5b).

**Table 5.** *
P. aeruginosa* proteomic expression *in vivo* in comparison to *in vitro* conditions Square colour indicates protein expression: up-regulation (red), down-regulation (green), undetermined (light grey). (a) Protein expression in *P. aeruginosa* populations from CF sputa, in comparison to populations grown *in vitro* [101]. The 15 proteins identified with a convergent pattern within the most samples were selected. (b) Protein expression in *P. aeruginosa* CF isolates in comparison to PAO1 determined *in vitro* in minimal medium M9 [99], rich medium LB [98, 102, 103] or in sputum-like media SCFM [99] or ASMDM (artificial sputum medium with high molecular mass DNA and mucin) [103], for the 15 proteins identified as expressed *in vivo*. NA, Not available.

Protein name	PAO1 locus	Product	(a) *In vivo* vs *in vitro*			(b) *In vitro* vs PA01		
			Expression in CF sputa	No. of samples with convergent pattern	No. of samples with detected protein	Expression in minimal medium	Expression in rich medium	Expression in sputum-like media
OprD	*PA0958*	Outer-membrane porin precursor		20	25			
OprH	*PA1178*	PhoP/Q and low Mg^2+^ inducible outer-membrane protein H1 precursor		27	33			
PA1288	*PA1288*	Probable outer-membrane protein precursor		26	33			
OprI	*PA2853*	Outer-membrane lipoprotein OprI precursor		26	35			
AlgE	*PA3544*	Alginate production outer-membrane protein AlgE precursor		20	21			
FumC1	*PA4470*	Fumarate hydratase		24	30			
PhuR	*PA4710*	Haem/haemoglobin uptake outer-membrane receptor precursor		22	32			
PA4793	*PA4793*	Hypothetical protein		23	31			
PA4837	*PA4837*	Probable outer-membrane protein precursor		28	31			
Hfq	*PA4944*	Hfq		19	29			
PstS	*PA5369*	Phosphate ABC transporter, periplasmic phosphate-binding protein		25	26			
NA	NA	TonB-dependent receptor		24	25			
Icd	*PA2623*	Isocitrate dehydrogenase		21	30			
RpsB	*PA3656*	30S ribosomal protein S2		20	28			
RplS	*PA3742*	50S ribosomal protein L19		23	26			

### Convergent phenotypes are selected by *P. aeruginosa* adaptation

**Fig. 2.**
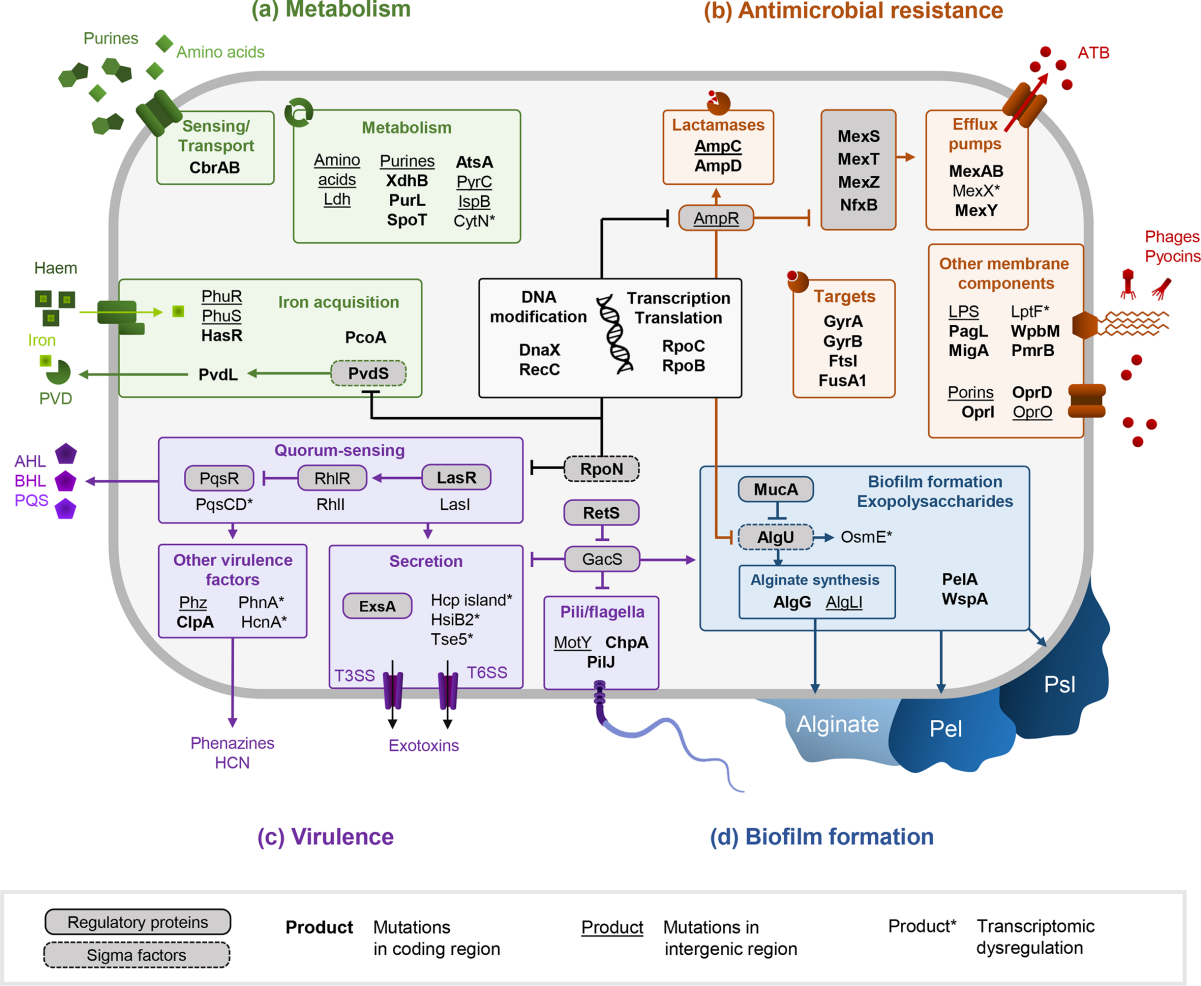
Pathways related to metabolism (**a**), antimicrobial resistance (**b**), virulence (**c**) and biofilm formation (**d**) altered during *P. aeruginosa* adaptation to the CF environment. DNA sequences of products in bold have been shown to accumulate non-synonymous mutations. Intergenic regions surrounding products that are underscored are mutated. Late isolates present a convergent transcriptomic dysregulation of the products marked by asterisks in comparison to early isolates.

As a result of the diversification of genetic, transcriptomic and proteomic profiles, CF-adapted *P. aeruginosa* can present various phenotypic signatures (Fig. 2) [46, 47, 104]. Although these are often found to be patient dependent [17, 105], similar phenotypes are frequently observed in adapted *P. aeruginosa* isolates, including alterations of metabolism, antibiotic resistance, biofilm and virulence. These phenotypes are associated with chronic infections as they promote bacterial persistence within lungs and have been extensively described [46, 47, 104, 106–108]. Interestingly, an analogous phenotypic diversification could be recently reproduced *in vitro* by experimental evolution in CF-mimicking conditions. Schick and colleagues observed that the complexity and the viscosity of the synthetic cystic fibrosis sputum medium (SCFM) containing mucin was sufficient to induce several common phenotypes of CF strains, such as antibiotic resistance, biofilm formation, loss of motility and production of virulence factors [109].

#### Metabolic alterations

The energetic metabolism of *P. aeruginosa* is largely affected by its adaptation to the CF environment. As a consequence of non-synonymous mutations in numerous metabolism-related genes, adapted *P. aeruginosa* strains present a differential and adjusted assimilation of the nutrients present in the CF lung (Fig. 2a) [17, 67, 105, 107, 110]. Auxotrophy or reduction of catabolic capacities are frequently observed and arise from either low or high molecule availability in the CF environment. Amino acid auxotrophy often arises in CF-adapted *P. aeruginosa* due to the high abundance of these molecules in CF sputum [107, 110–112]; in addition, purine auxotrophy can be established in DNA-rich sputa [113]. Development of new metabolic capacities can nonetheless arise through enrichment of the accessory genome in metabolic functions [17, 28, 60]. This adjusted metabolism increases *P. aeruginosa* fitness in the CF environment, but it often results in a slowed growth in laboratory conditions in comparison to non-adapted isolates [7, 8, 12, 13, 18, 107, 110, 114]. This modification of metabolic activities can limit effective detection and treatment of infecting *P. aeruginosa *, as illustrated by the emergence of highly resistant small colony variants (SCVs) and viable but non-culturable (VBNC) isolates [115–117].

#### Antimicrobial resistance and biofilm

Another feature limiting treatment of *P. aeruginosa* infection is the development of resistance mechanisms to antimicrobials. In comparison to early strains, late *P. aeruginosa* isolates present a greater antibiotic resistance acquired through different mechanisms: (i) alteration of antibiotic transport, (ii) increase of antibiotic degradation, and (iii) alteration of antibiotic targets [118]. The alteration of antibiotic transport is characterized by a decrease of antibiotic input through reduction of porin activities, and in an increase of drug output through modification of the efflux pumps activity. Particularly, *oprD* repression and *mexAB* overexpression, induced by mutations in their own coding sequences or in their regulators, are frequently responsible for β-lactam resistance in CF *P. aeruginosa* (Fig. 2b) [10, 118, 119]. Such resistance can also be promoted by the genome enrichment of accessory genes involved in multidrug secretion. The many transporters constituting the accessory genome of the LES epidemic strain, thus, contribute to its high antibiotic resistance and its epidemiological success [23]. The increase in antibiotic degradation is mainly perpetrated by an overproduction of the cephalosporinase AmpC, induced by mutations in the *ampCD* genes but also in the coding sequencing of their regulator AmpR (Fig. 2b) [118]. Finally, the increase of *P. aeruginosa* multidrug resistance can also involve the alteration of several antibiotic targets, such as the DNA gyrase GyrAB, the penicillin-binding protein FtsI or the lipopolysaccharide (LPS) of the bacterial outer membrane [11, 118, 120, 121] (Fig. 2b). The latter undergoes important alterations of its three components during *P. aeruginosa* adaptation to the CF environment. Mutations in *pmrB*, *migA* and *pagL* are associated with structural modifications of the lipid A part of the LPS, inducing resistance to polymyxins [10–12, 120, 122]. The alteration of MigA and LptF can also affect the synthesis of the core oligosaccharide and the transport of the mature LPS, although their impact on antibiotic resistance remains poorly understood [121, 123, 124]. Finally, CF isolates often lack the O-antigen polysaccharide of the LPS due to mutations in *wbp* genes, resulting in lower virulence and increased tolerance to gentamicin [121, 125].

Besides antibiotics, LPS modifications also affect *P. aeruginosa* resistance to phages and bacteriocins [120]. In CF-adapted *P. aeruginosa *, mutations in LPS biosynthesis genes were shown to decrease phage susceptibility by hampering LPS-mediated recognition [120, 126]. In contrast, chronic CF isolates are often more susceptible to the *P. aeruginosa *-produced bacteriocins, pyocins, due to an improved access to the cell envelope following the structural alterations of the O-antigen [120, 127, 128]. However, pyocin production is also frequently reduced in chronic CF *P. aeruginosa* [126, 127].

Resistance to antimicrobials is also associated with an increased formation of biofilm. The exopolysaccharide matrix, constituted of varying proportions of Pel, Psl or alginate molecules according to the strain, indeed allows the constitution of a physical and chemical barrier against antimicrobials (Fig. 2d) [129–132]. CF-adapted strains often present an up-regulation of Pel, Psl and/or alginate exopolysaccharides production; hence, increasing biofilm formation, modifying the composition of its matrix and favouring antimicrobial resistance [130]. Pel and Psl overproduction is, thus, responsible for the persistence phenotype of rugose small colony variants (RSCVs) in CF *P. aeruginosa* [133, 134]. Mucoid isolates, mainly arising from *mucA* alterations inducing alginate overproduction, are also associated with poorer clinical outcome and greater inflammation [135–138]. Interestingly, mucoid and non-mucoid isolates are often co-isolated from CF patients, due to diversification or reversion of the phenotype through compensatory mutations, in *algU* for instance (Fig. 2d) [11, 18, 72]. Sessile lifestyle is also promoted by a loss of motility linked to inhibition of pili and flagella synthesis [10, 12, 18]. Alterations of these membrane components, as well as LPS modification and biofilm formation, reduce the induction of the host inflammasome and, thus, efficient bacterial elimination from the lungs [106, 108].

#### Virulence

In the same way, *P. aeruginosa *-adapted isolates have been shown to secrete fewer virulence factors, which are both immunogenic and costly to produce [10, 18, 108]. Iron plays a pivot role in bacterial virulence and its acquisition is affected during *P. aeruginosa* adaptation to the CF environment. Alteration of pyoverdine siderophore synthesis through mutations in the regulator *pvdS* and the *pvd* genes is often observed, inducing a loss of virulence [125, 139, 140]. In contrast, iron acquisition through haem is promoted in adapted isolates thanks to the up-regulation of Phu and Has systems (Fig. 2a) [139, 141]. Changes in the accessory genome composition also undoubtedly affect *P. aeruginosa* virulence, as chronic or eradicated CF isolates present a different repertory of accessory functions than virulent ones [17, 32]. Alteration of the genomic islands PAPI-1 and PAPI-2 and the LES phages can greatly lower *P. aeruginosa* virulence [21, 22, 24]. In connection with this, CF isolates from chronic infection strains often lacks the PAPI-2 encoded cytotoxin ExoU. They instead harbour the type III secretion system (T3SS) effector ExoS, which is chromosomally encoded and has less virulent properties than ExoU [142–145]. However, mutations in major virulence and quorum-sensing (QS) regulators, such as *retS*, *exsA* or *lasR*, are the main perpetrators of the low-virulence state of chronic *P. aeruginosa* (Fig. 2c).

#### QS rewiring and modification of microbial interactions

The alterations of QS systems suggest that *P. aeruginosa* adaptation goes along with a reduction of social behaviours. This hypothesis is supported by the high frequency of *lasR* mutations that are also acquired during *in vitro* evolution of *P. aeruginosa* [146, 147]. On the one hand, the emergence of *lasR*-mutant social cheaters within the bacterial population suggest a loss of intra-species cooperative behaviours as these mutants will benefit from extracellular factors produced by other members without paying the energy cost [148–150]. However, this also indicates that QS activities and social behaviours need to be considered at the whole population scale. On the other hand, several recent studies depicted that *lasR* mutants isolated from CF infections retained an active QS through a *lasR*-independent induction of the Rhl system. This phenomenon was often related to compensatory mutations in the pathoadaptive *mexT* gene [63–66], and not by alteration of *rhl* genes. The latter are indeed rarely mutated during *P. aeruginosa* evolution within CF lungs, underscoring the importance of maintaining a functional Rhl system during chronic infections. Instead of a loss of QS, *P. aeruginosa* adaptation to the CF lung rather induces a rewiring of QS networks for the benefit of a Rhl-mediated social behaviour within the bacterial population. Furthermore, the intra-species interactions of *P. aeruginosa* do not seem to involve pyocins anymore, since both pyocin resistance and production are frequently reduced in chronic isolates [120, 126, 127]. However, pyocins and many of the QS-regulated factors also play a critical role in interspecies interactions, such as the type VI secretion system (T6SS) and pyocyanin (Fig. 2c) [151–155]. And indeed, an increasing number of studies highlight an evolution of *P. aeruginosa* interactions with other co-colonizing micro-organisms in the CF environment [155–160].

## Concluding remarks and future perspectives

The numerous sequencing studies performed on clinical isolates allowed the description of the main genetic mechanisms of *P. aeruginosa* adaptation to the CF environment. This adaptation mainly relies on the accumulation and the selection of small mutations in pathoadaptive genes. For the first time, this phenomenon was recently shown to occur within intergenic regions as well. As these non-coding elements were rarely taken into account in genomic studies, reanalyses of the vast amount of sequencing data already available should allow a better examination of their role in the *P. aeruginosa* adaptation process. At the same time, the ambiguous impact of recombination and large chromosomal rearrangements on pathoadaptation could be clarified by combining second- and third-generation sequencing methods to assemble complete genomes.

Alteration of pathoadaptive elements allows the establishment of persistence phenotypes in *P. aeruginosa *, such as high antibiotic resistance through an increased efficiency of antimicrobial efflux, an enhanced ability to form biofilm and a slowed metabolism. In addition, the low-virulence state of CF-adapted *P. aeruginosa* limits the proper functioning of the host immune responses. However, the precise relationship between these phenotypes and the *P. aeruginosa* genotype remains difficult to evaluate, especially due to the intense diversification occurring during adaptation and the pleiotropic effects of most mutations. The study of several isolates per time point throughout longitudinal studies would allow a better overview of the different evolutionary paths taken by the bacterium within CF lungs. Assessing the changes in gene and protein expressions during *P. aeruginosa* adaptation thanks to -omics methods can also address some of these issues, with particular attention to the expression conditions. Transcriptomic and proteomic studies *in vivo* or in CF-like conditions, thus, appears essential to gain more insight in the physiological adaptation of *P. aeruginosa* to the CF environment.

The description of *P. aeruginosa* adaptive process ensures a better understanding of the selection forces that drive its evolution within the CF lung. While some of them are already known, such as antibiotic and oxidative stresses, other selective pressures remain little explored. Due to the polymicrobial nature of CF infections, the role of other microbial communities in *P. aeruginosa* adaptive mechanisms deserves more consideration. The activities of native or co-colonizing micro-organisms can deeply affect the environment characteristics, such as the distribution and availability of nutrients, iron or antimicrobial molecules. Moreover, a range of microbial interactions can either limit or promote *P. aeruginosa* persistence and, thus, adaptation within CF lung infections [156–158, 160–162]. In line with this, the presence of *Staphylococcus aureus* has been shown to promote *P. aeruginosa* colonization [163], whereas the latter was negatively associated with infection by other pathogens such as *Burkholderia cepacia* and *Stenotrophomonas maltophilia* [164]. Besides pathogens, the role of the normal lung microbiota is increasingly considered since commensal anaerobes have been shown to impact the antibiotic resistance and virulence of *P. aeruginosa* [161, 162]. Thus, the presence of these micro-organisms may influence establishment and adaptation of *P. aeruginosa* in the CF environment. Ultimately, the comprehensive understanding of this adaptation appears pivotal to limit the establishment of chronic *P. aeruginosa* infections.

## Supplementary Data

Supplementary material 1
