# Supplementary material for: From genotype to phenotype: adaptations of Pseudomonas aeruginosa to the cystic fibrosis environment
Source: Microb Genom. 2021 Feb 2;7(3):mgen000513. doi: 10.1099/mgen.0.000513 (PMC8190622; doi:10.1099/mgen.0.000513)
Supplement: Supplementary material 1 [file mgen-7-0513-s001.pdf]

**Table S1: *P. aeruginosa* intergenic regions under positive selection.** Mutations in intergenic regions were identified as positively selected by Khademi *et al* . (81) and sorted in this table according to the index of interest (Column D). For each intergenic region, the index was calculated by multiplying the number of intergenic mutations (Column A) by the number of impacted lineages (Column B) and the number of independent studies identifying the region as mutated (Column C).

| | | | | A | B | C | D | |
|---|---|---|---|---|---|---|---|---|
| **Name of up-stream gene** | **Name of down-stream gene** | **PAO1 locus** | **Product** | **No. of intergenic mutation** | **No. of lineages with mutated isolate** | **No. of independent studies identifying the region as mutated** | **Index of interest** (Column A x Column B x Column C) | **References** |
| *phuS* | *phuR* | PA4709 - PA4710 | PhuS//Heme/Hemoglobin uptake outer membrane receptor PhuR precursor | 40 | 4 | 4 | **640** | 7,9,18,81 |
| | | PA0428 - PA0429 | probable ATP-dependent RNA helicase//hypothetical protein | 34 | 10 | 1 | **340** | 81 |
| | | PA4786 - PA4787 | probable short-chain dehydrogenase//probable transcriptional regulator | 28 | 12 | 1 | **336** | 81 |
| | | PA4690.5 - PA4691 | 16S ribosomal RNA//hypothetical protein | 54 | 6 | 1 | **324** | 81 |
| | | PA2535 - PA2536 | probable oxidoreductase//probable phosphatidate cytidylyltransferase | 18 | 6 | 2 | **216** | 7,81 |
| *motY* | *pyrC* | PA3526 - PA3527 | probable outer membrane protein precursor//dihydroorotase | 32 | 6 | 1 | **192** | 81 |
| | | PA3230 - PA3231 | conserved hypothetical protein//downstream hypothetical protein | 24 | 7 | 1 | **168** | 81 |
| *algL* | *algI* | PA3547 - PA3548 | poly(beta-d-mannuronate) lyase precursor AlgL//alginate o-acetyltransferase AlgI | 14 | 6 | 2 | **168** | 7,81 |
| | | PA0976.1 - PA0977 | tRNA-Lys//hypothetical protein | 26 | 6 | 1 | **156** | 81 |
| *rplU* | *ispB* | PA4568 - PA4569 | 50S ribosomal protein L21//octaprenyl-diphosphate synthase | 22 | 7 | 1 | **154** | 81 |
| *phzM* | *phzA1* | PA4209 - PA4210 | probable phenazine-specific methyltransferase//probable phenazine biosynthesis protein | 12 | 6 | 2 | **144** | 7,81 |
| *oprO* | | PA3280 - PA3281 | Pyrophosphate-specific outer membrane porin OprO precursor//hypothetical protein | 10 | 5 | 2 | **100** | 7,81 |
| *ldh* | | PA3418 - PA3419 | leucine dehydrogenase//hypothetical protein | 10 | 5 | 2 | **100** | 7,81 |
| *ampR* | *ampC* | PA4109 - PA4110 | transcriptional regulator AmpR//beta-lactamase precursor | 12 | 4 | 2 | **96** | 9,81 |
| | *rmlB* | PA5160.1 - PA5161 | tRNA-Thr//dTDP-D-glucose 4,6-dehydratase | 16 | 6 | 1 | **96** | 81 |
| | | PA1334 - PA1335 | probable oxidoreductase//probable two-component response regulator | 28 | 3 | 1 | **84** | 81 |
| | | PA0979 - PA0980 | conserved hypothetical protein//hypothetical protein | 16 | 5 | 1 | **80** | 81 |
| *ndvB* | | PA1163 - PA1164 | NdvB//conserved hypothetical protein | 10 | 4 | 2 | **80** | 7,81 |
| *norM* | | PA1361 - PA1362 | NorM//hypothetical protein | 10 | 4 | 2 | **80** | 7,81 |
| | | PA3965 - PA3966 | probable transcriptional regulator//hypothetical protein | 16 | 5 | 1 | **80** | 81 |
| | | PA1941 - PA1942 | hypothetical protein//hypothetical protein | 12 | 6 | 1 | **72** | 81 |
| *etfB* | | PA2952 - PA2953 | electron transfer flavoprotein beta-subunit//electron transfer flavoprotein-ubiquinone oxidoreductas | 14 | 5 | 1 | **70** | 81 |
| | *aph* | PA4118 - PA4119 | hypothetical protein//aminoglycoside 3'-phosphotransferase type IIb | 14 | 5 | 1 | **70** | 81 |
| *lptD* | | PA0595 - PA0596 | LPS-assembly protein LptD//hypothetical protein | 16 | 4 | 1 | **64** | 81 |
| *pdxB* | *aceK* | PA1375 - PA1376 | erythronate-4-phosphate dehydrogenase//isocitrate dehydrogenase kinase/phosphatase | 12 | 5 | 1 | **60** | 81 |
| | | PA1841 - PA1842 | hypothetical protein//hypothetical protein | 12 | 5 | 1 | **60** | 81 |
| *xthA* | | PA2545 - PA2546 | exodeoxyribonuclease III//probable ring-cleaving dioxygenase | 20 | 3 | 1 | **60** | 81 |
| *moaC* | | PA3918 - PA3919 | molybdopterin biosynthetic protein C//conserved hypothetical protein | 12 | 5 | 1 | **60** | 81 |
| | | PA4960 - PA4961 | probable phosphoserine phosphatase//hypothetical protein | 12 | 5 | 1 | **60** | 81 |
| *poxB* | | PA5297 - PA5298 | pyruvate dehydrogenase (cytochrome)//xanthine phosphoribosyltransferase | 12 | 5 | 1 | **60** | 81 |
| | | PA0588 - PA0589 | conserved hypothetical protein//conserved hypothetical protein | 14 | 4 | 1 | **56** | 81 |
| | | PA0977 - PA0978 | hypothetical protein//conserved hypothetical protein | 14 | 4 | 1 | **56** | 81 |
| | | PA1191 - PA1192 | hypothetical protein//conserved hypothetical protein | 14 | 4 | 1 | **56** | 81 |
| *ppc* | | PA3687 - PA3688 | phosphoenolpyruvate carboxylase//hypothetical protein | 14 | 4 | 1 | **56** | 81 |
| | | PA1348 - PA1349 | hypothetical protein//conserved hypothetical protein | 10 | 5 | 1 | **50** | 81 |
| | *bacA* | PA1958 - PA1959 | probable transporter//bacitracin resistance protein | 16 | 3 | 1 | **48** | 81 |
| | | PA3779 - PA3780 | hypothetical protein//putative TRAP-type C4-dicarboxylate transport | 12 | 4 | 1 | **48** | 81 |
| | | PA3785 - PA3786 | conserved hypothetical protein//hypothetical protein | 8 | 3 | 2 | **48** | 7,81 |
| | | PA4792 - PA4793 | conserved hypothetical protein//hypothetical protein | 16 | 3 | 1 | **48** | 81 |
| | | PA5491 - PA5492 | probable cytochrome//conserved hypothetical protein | 8 | 3 | 2 | **48** | 7,81 |
| | | PA0980 - PA0981 | hypothetical protein//hypothetical protein | 14 | 3 | 1 | **42** | 81 |
| | | PA3341 - PA3342 | probable transcriptional regulator//hypothetical protein | 14 | 3 | 1 | **42** | 81 |
| | *phrD* | PA0714 - PA0714.1 | hypothetical protein//PhrD | 10 | 4 | 1 | **40** | 81 |

| | | | | | | | | |
|---|---|---|---|---|---|---|---|---|
| | | PA1243 - PA1244 | probable sensor/response regulator hybrid//hypothetical protein | 10 | 4 | 1 | **40** | 81 |
| *opdT* | | PA2505 - PA2506 | tyrosine porin OpdT//hypothetical protein | 10 | 4 | 1 | **40** | 81 |
| | | PA4089 - PA4090 | probable short-chain dehydrogenase//hypothetical protein | 10 | 4 | 1 | **40** | 81 |
| | | PA0574.1 - PA0575 | tRNA-Met//conserved hypothetical protein | 12 | 3 | 1 | **36** | 81 |
| *tyrZ* | | PA0668 - PA0668.1 | tyrosyl-tRNA synthetase 2//16S ribosomal RNA | 18 | 2 | 1 | **36** | 81 |
| | *ccoP1* | PA1551 - PA1552 | probable ferredoxin//Cytochrome c oxidase, cbb3-type, CcoQ subunit | 12 | 3 | 1 | **36** | 81 |
| | *hisF1* | PA5139 - PA5140 | hypothetical protein//imidazoleglycerol-phosphate synthase, cyclase subunit | 12 | 3 | 1 | **36** | 81 |
| *plsB* | | PA3673 - PA3674 | glycerol-3-phosphate acyltransferase//hypothetical protein | 6 | 3 | 2 | **36** | 7,81 |
| *cerN* | | PA0845 - PA0846 | CerN//probable sulfate uptake protein | 10 | 3 | 1 | **30** | 81 |
| *ctpH* | | PA2561 - PA2562 | probable chemotaxis transducer//hypothetical protein | 10 | 3 | 1 | **30** | 81 |
| | *guaA* | PA3768 - PA3769 | probable metallo-oxidoreductase//GMP synthase | 10 | 3 | 1 | **30** | 81 |
| | | PA1142 - PA1143 | probable transcriptional regulator//hypothetical protein | 8 | 3 | 1 | **24** | 81 |
| *nagZ* | *psrA* | PA3005 - PA3006 | beta-N-acetyl-D-glucosaminidase//transcriptional regulator PsrA | 8 | 3 | 1 | **24** | 81 |
| *gshB* | *pilG* | PA0407 - PA0408 | glutathione synthetase//twitching motility protein PilG | 6 | 3 | 1 | **18** | 81 |
| | *plcR* | PA0842 - PA0843 | glycosyl transferase//phospholipase accessory protein PlcR precursor | 6 | 3 | 1 | **18** | 81 |
| *popD* | *exsC* | PA1709 - PA1710 | Translocator outer membrane protein PopD precursor//ExsC, exoenzyme S synthesis protein C pr | 6 | 3 | 1 | **18** | 81 |
| *hmgA* | | PA2009 - PA2010 | homogentisate 1,2-dioxygenase//probable transcriptional regulator | 6 | 3 | 1 | **18** | 81 |
| | | PA2069 - PA2070 | probable carbamoyl transferase//hypothetical protein | 6 | 3 | 1 | **18** | 81 |
| | | PA2418 - PA2419 | hypothetical protein//probable hydrolase | 6 | 3 | 1 | **18** | 81 |
| *phzG1* | *phzS* | PA4216 - PA4217 | probable pyridoxamine 5'-phosphate oxidase//flavin-containing monooxygenase | 6 | 3 | 1 | **18** | 81 |
| | | PA4873 - PA4874 | probable heat-shock protein//conserved hypothetical protein | 6 | 3 | 1 | **18** | 81 |
| *nasS* | *acnB* | PA1786 - PA1787 | NasS//aconitate hydratase 2 | 10 | 1 | 1 | **10** | 81 |
| | *treA* | PA2415 - PA2416 | hypothetical protein//periplasmic trehalase precursor | 8 | 1 | 1 | **8** | 81 |
| *rluC* | *rne* | PA2975 - PA2976 | ribosomal large subunit pseudouridine synthase C//ribonuclease E | 8 | 1 | 1 | **8** | 81 |
| | | PA0014 - PA0015 | hypothetical protein//hypothetical protein | 6 | 1 | 1 | **6** | 81 |
| | | PA1013.1 - PA1014 | tRNA-Ser//probable glycosyl transferase | 6 | 1 | 1 | **6** | 81 |
| *hcnC* | | PA2195 - PA2196 | hydrogen cyanide synthase HcnC//probable transcriptional regulator | 6 | 1 | 1 | **6** | 81 |
| | | PA2480 - PA2481 | probable two-component sensor//hypothetical protein | 6 | 1 | 1 | **6** | 81 |
| | *apeA* | PA2855 - PA2856 | hypothetical protein//lysophospholipase A | 6 | 1 | 1 | **6** | 81 |
| | *pyrF* | PA2875 - PA2876 | conserved hypothetical protein//orotidine 5'-phosphate decarboxylase | 6 | 1 | 1 | **6** | 81 |
| *rsmZ* | *rpoS* | PA3621.1 - PA3622 | regulatory RNA RsmZ//sigma factor RpoS | 6 | 1 | 1 | **6** | 81 |
| | | PA4040 - PA4041 | hypothetical protein//hypothetical protein | 6 | 1 | 1 | **6** | 81 |
| | | PA4837 - PA4838 | probable outer membrane protein precursor//hypothetical protein | 6 | 1 | 1 | **6** | 81 |
| | *polA* | PA5492 - PA5493 | conserved hypothetical protein//DNA polymerase I | 6 | 1 | 1 | **6** | 81 |
